# Bone Health in a Nonjaundiced Population of Children with Biliary Atresia

**DOI:** 10.1155/2009/387029

**Published:** 2009-07-05

**Authors:** Rachel A. Kramer, Babette S. Zemel, Jessica L. Arvay-Nezu, Virginia A. Stallings, Mary B. Leonard, Barbara A. Haber

**Affiliations:** ^1^The Division of Gastroenterology, Hepatology & Nutrition, Children's Hospital of Philadelphia, PA 19104, USA; ^2^The Division of Nephrology, Children's Hospital of Philadelphia, PA 19104, USA

## Abstract

*Objectives*. To assess bone health in a cohort of nonjaundiced children with biliary atresia (BA) and the effect of growth and development on bone outcomes. *Methods*. Children ages one to eighteen years receiving care from Children's Hospital of Philadelphia were recruited. Each child was seen once and assessed for growth, pubertal development, concurrent medications, bilirubin, ALT, albumin, vitamin D status, bone mineral density (BMD), and bone mineral content (BMC) of the lumbar spine and whole body. *Results*. BMD declined significantly with age, and upon further analysis with a well-phenotyped control cohort, it was found that BMC was significantly decreased for both lumbar spine and whole body, even after adjustment for confounding variables. An age interaction was identified, with older subjects having a significantly greater impairment in BMC. *Conclusions*. These preliminary results demonstrate that children with BA, including those without jaundice, are likely to have compromised bone health even when accounting for height and puberty, which are common confounding factors in chronic disease. Further investigation is needed to identify the determinants of poor bone mineral status and to develop strategies to prevent osteoporosis later in life.

## 1. Introduction

Biliary atresia (BA) is an infantile liver disease resulting in a progressive fibro-obliteration of the biliary tree [1]. Successful surgical hepatoportoenterostomy promotes biliary drainage, but does not halt the hepatic inflammatory process [2]. Follow-up care includes serological assessment of both total and direct bilirubin and alanine transferase, which are surrogate markers of cholestasis and inflammation, respectively. The approach to clinical management is to optimize growth and nutrition, yet rarely is bone health assessed unless there is presence of jaundice, a history of fracture, inadequate vitamin D repletion, or exposure to medications that may affect bone health such as corticosteroids.

Bone disease among patients with liver problems is most frequently discussed in the context of chronic cholestasis and fat-soluble vitamin deficiency [3–8]. However, it is now known that not only malabsorption of vitamin D and calcium [3–10], but also a host of cytokines [6, 9, 11–13] can contribute to bone loss. A number of other inflammatory diseases such as inflammatory bowel disease [14–18], rheumatoid arthritis [19], juvenile idiopathic arthritis [20], and systemic lupus erythematosus [21], have documented low bone mineral density and, in some studies, resultant osteopenia and osteoporosis. Since BA is an inflammatory disorder [1, 22], with ongoing issues of malabsorption present even when surgery is successful, it is postulated that bone health might be compromised even when jaundice is not present.

This preliminary study assessed bone health in a group of nonjaundiced children with BA from ages one to eighteen years. Since growth and maturation may be compromised in a chronic disease such as BA, we used normative data and an analysis that accounted for confounding variables of height and puberty as well as race and gender.

## 2. Materials and Methods

### 2.1. Study Subjects

All subjects with BA had undergone a Kasai procedure and none had received a liver transplant at the time of enrollment. In addition, none of the BA subjects had received steroids post-Kasai. Subjects were excluded if they had any other chronic illness known to affect growth, pubertal development or bone health such as renal insufficiency, thyroid disease, parathyroid disease, or growth hormone insufficiency. Hologic control data was used for the cohort of 16. However, due to limitations of that control data (not controlled for ethnicity or tanner) we also performed an analysis using a well-studied local cohort of 178 healthy control subjects, ages 4 to 18 years, recruited from the pediatric clinics and surrounding community. Subjects with medical conditions or medications potentially affecting growth and development were excluded. Informed consent was obtained from each subject's legal guardian prior to the study, and assent was obtained from subjects aged six years and older. The protocol was approved by the CHOP Institutional Review Board.

### 2.2. Height, Weight, and Pubertal Assessment

All subjects were evaluated in the Nutrition and Growth Laboratory. On the day of the DXA measurement, height and weight measurements were obtained in triplicate using a wall-mounted stadiometer and digital scale, respectively; the mean of the three measures was used in the analysis. Pubertal status was evaluated by physical exam and classified according to the method of Tanner [23]. Age and gender specific standard deviation scores (z-scores) for height (HAZ), weight (WAZ) and body mass index (BMIZ) were calculated using the Center for Disease Control 2000 Growthcharts [24]. 

### 2.3. Laboratory and Bone Age Assessment

For the BA group, fasting samples were obtained for total bilirubin, direct bilirubin, albumin, alanine transferase (ALT), and 25–OH vitamin D levels (EXOTERIX INC. Laboratory Services, competitive protein binding assay). Bone age was obtained on the same day as the DXA scan, and scored according to the Tanner-Whitehouse 2 (TW2) method [25]. A single observer scored all radiographs of the left hand and wrist. Three diet records were assessed for calcium intake.

### 2.4. DXA Measures

Bone mass in the posterio-anterior lumbar spine (*L*_1–4_) and whole body was measured by DXA (Hologic QDR 2000, Waltham, Mass, USA) with a fan beam in the array mode. All subjects were measured on the same machine. The measurements were performed using standard positioning techniques. Quality control scans were performed daily using a simulated *L*_1–4_ lumbar spine made of hydroxyapatite encased in epoxy resin. In our institution, the in vitro coefficient of variation was less than 0.6% and the in vivo coefficient of variation in adults was less than 1%. 

The DXA scans were analyzed to generate measures of vertebral projected bone area (cm^2^), BMC (g) and areal bone mineral density (BMD, g/cm^2^). Because the standard software for the analysis of the lumbar spine frequently fails to detect a complete bone map in children, all lumbar spine scans were analyzed with the low-density software, as previously described [26]. 

### 2.5. Statistics

The BMC of children with BA were evaluated relative to the healthy control group. Mean differences in the demographic and anthropometric outcomes between BA subjects and controls were assessed using the *t*-test. Two-sided tests of hypotheses were used and a *P*-value <.05 was considered significant. The race and gender distributions were compared using the Chi-square test. 

Due to inadequacies of Hologic normative data for this generation machine [27], we recruited our own separate control cohort and investigated BMC. The primary outcomes were BMC of the spine and whole body, less the head. Assessment of BMC in children and adolescents required adjustment for age and body size. The relationship between BMC and age or body size is nonlinear and heteroscedastic. Therefore, all continuous variables were natural log transformed for multiple regression analysis to assess differences between the BA and control groups adjusting for important covariates. A polynomial term for age was added to the models since the natural log transformation did not account for the non-linear increase in BMC with age. Dummy variables for black ethnicity and female gender were included in the models because of the known BMC differences between blacks versus nonblacks and boys versus girls. Tanner stage was included in the models if it was statistically significant. A series of sequential regression analyses was performed to evaluate differences in BMC between the BA and control groups when accounting for the effects of (1) gender, black ethnicity and age, (2) same as the previous model, plus height and Tanner stage (if statistically significant), and (3) same as the previous model plus an interaction term (biliary atresia *x* age) to test for age-related declines in BMC status in the BA group after accounting for other important covariates. The effect of BA in each multivariate model is presented in the text as the adjusted ratio of the outcome measure (e.g., lumbar spine BMC or whole body BMC) divided by the outcome measure in the controls, with 95 percent confidence intervals. The adjusted ratio and confidence intervals were calculated as the exponentiated estimates of the regression variables for BA.

The difference between the BA and control group was expressed as the adjusted ratio of the outcome measure in the subjects with BA divided by the outcome measure in the control subjects, with 95% confidence intervals. The adjusted ratio and confidence intervals were calculated as the exponentiated estimates of the regression parameters.

The regression models described above (Table 2, model 2) were used to generate standardized residuals for whole body and lumber spine BMC for the subjects in the BA group. The standardized residuals were used to represent standard deviation scores, similar to *Z*-scores, for BMC adjusted for sex, black ethnicity, age, height, spine BMC, and Tanner stage.

Spearman tests were performed to examine the correlation of serologic tests (total bilirubin, direct bilirubin, and alanine transferase) and BMC standardized deviation score of both the spine and whole body (Table 3).

## 3. Results

### 3.1. Subject Characteristics

**Figure 1:**
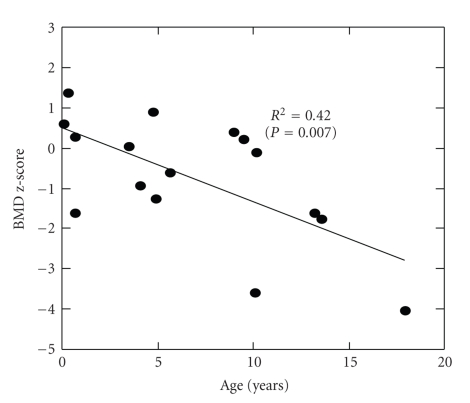
BMD *Z*-Score versus age.

A total of 16 BA patients were enrolled in the study. Anthropometric measurements of height and weight were compared to the U.S. Reference Data set (CDC 2000). Although mild stunting was present as indicated by the mean height-for-age z-score (HAZ) of −0.31, the weight age z-score (WAZ) and body mass index z-score (BMIZ) were essentially normal. Eight of 16 subjects received vitamin D supplementation prescribed by their primary liver physician. No significant difference in mean vitamin D level was found between those on supplements and those not. All subjects had normal 25–OH vitamin D levels, with a mean of 24.3 ng/mL and none of the subjects were frankly deficient (<10 ng/mL). Calcium intake based on three diet records analyzed by Food Processor ranged from 90% to 210% of for age-based comparisons. No significant bone age delay existed. Linear regression analyses were conducted to compare the BMD in BA subjects to the Hologic age-matched normative data. The linear regression line of BMD z-scores versus age showed that 42% of the variance was explained by age (*P* = .0007), suggesting a decline in BMD with age (Figure 1).

We explored the relationship of confounding variables on our finding using a carefully phenotyped control cohort that included ethnicity and tanner. A total of 11 patients with BA were studied in this cohort. The 5 youngest patients were excluded due to the age range of the control cohort. Characteristics of the BA subjects are shown in Table 1. Mild stunting was present, as indicated by the mean height-for-age z-score (HAZ) of −0.11, while the body mass index z-score (BMIZ) was in the normal range. Compared to controls, BA subjects' HAZ and BMIZ were not significantly different. Both mean age and percentage African American subjects were less in the BA group but these differences were only significant for the percentage African American subjects (*P* = .04). For the BA group (*n* = 6), the mean 25–OH vitamin D concentration was 26 ng/mL, and none of the subjects were frankly deficient (<10 ng/mL). Only one of eleven subjects had a total bilirubin level greater than 1.00 mg/dL and that subject had a direct bilirubin of 0.00 mg/dL. The mean direct bilirubin level for the cohort of BA subjects was 0.18 mg/dL. No significant bone age delay existed and none of the subjects had a prior fracture. In addition, none of the subjects were receiving vitamin D supplementation.

### 3.2. DXA Results

**Figure 2:**
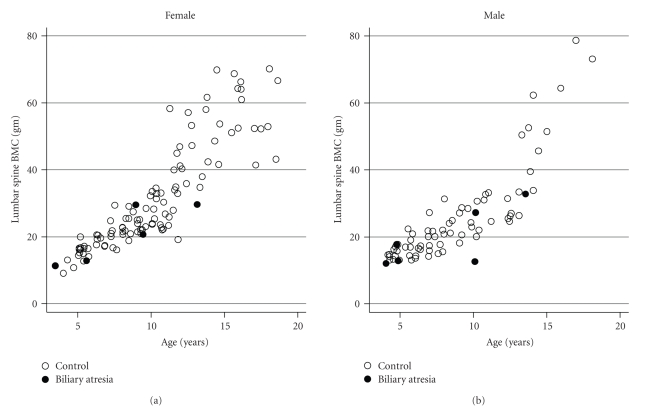
Age versus lumber spine bone mineral content in BA subjects compared to controls.

**Figure 3:**
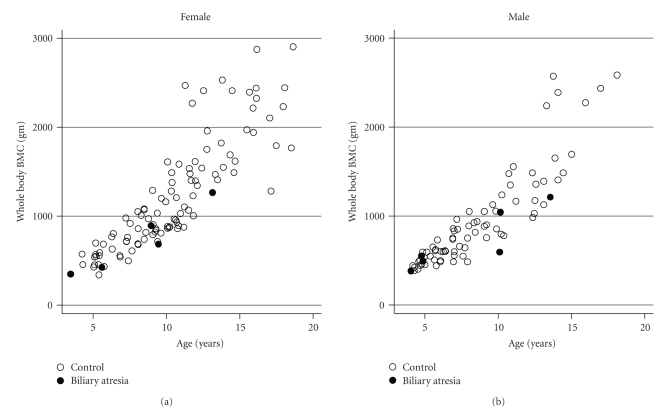
Age versus whole body bone mineral content in BA subjects compared to controls.

The distribution of BMC for age in BA subjects compared to controls is illustrated in Figures 2  and 3. The distribution suggests that low BMC status may be more likely to occur at older ages. It also illustrates the nonlinear and heteroscedastic distribution of BMC relative to age in growing children.

#### 3.2.1. Lumbar Spine BMC

The sequential regression models to evaluate BMC in BA compared to controls are shown in Table 2. Model 1 evaluated spine BMC adjusted for age, black ethnicity and gender. BMC was significantly reduced in subjects with BA. The adjusted ratio for spine BMC in subjects with BA compared with controls was 0.87 (95% C.I. 0.76, 0.98), *R* ^2^ = 0.82, *P* ≤ .001). However, the reduced BMC for age may be due to decreased height for age in the BA group, and subsequently smaller bones for age. Delayed puberty may also have an effect on BMC status. In model 2, height and Tanner stage were added to the model. Adjustment for these covariates attenuated the deficits in BMC and the explained variance (overall *R* ^2^) increased; however, the deficits remained significant. The fullyadjusted ratio of spine BMC in the BA subjects compared with controls was 0.88 (95% C.I. 0.80, 0.97, *R* ^2^ = 0.90, *P* < .001). That is, BMC was 12% lower, on average, in BA subjects compared with controls. Model 3 shows that the interaction term for BA *x * age was statistically significant and negative, indicating that the adverse effect of BA on lumbar spine BMC was greater at older ages. However, there was no appreciable increase in the overall *R* ^2^ for the model.

#### 3.2.2. Whole Body BMC

The initial model for whole body BMC shown in Table 2 evaluated whole body BMC in subjects with BA compared with controls, adjusted for age, gender and black ethnicity. The adjusted ratio for whole body BMC in subjects with BA compared with controls was 0.88 (95% C.I. 0.78, 1.00, *R* ^2^ = 0.85, *P* < .001). The model was then adjusted for height; Tanner stage was not statistically significant and was therefore excluded from this and subsequent models. Whole body BMC remained significantly decreased in the subjects with BA compared with control subjects: ratio 0.91 (95% C.I. 0.83, 0.99, *R* ^2^ = 0.93, *P* < .001). That is, whole body BMC was 9% lower on average, adjusted for height, age, gender, and race in the BA subjects compared with controls. Model 3 shows that the interaction term for BA *x * age was statistically significant and negative, indicating that the adverse effect of BA on whole body BMC was greater at older ages. However, there was only a modest increase in the overall *R* ^2^ for the model. 

Analyses of direct bilirubin, total bilirubin and alanine transferase were performed using Spearman tests to investigate correlations between these serologic tests and BMC standard deviation score. A significant negative correlation was identified for direct bilirubin level and spine BMC *z*-score and a trend toward a negative relationship was identified for whole body BMC *z*-score. (Table 3).

## 4. Discussion

These analyses demonstrate the existence of a significant bone mineral deficit in a cohort of nonjaundiced BA patients. In part, bone mineral deficits were due to smaller body size, but significant deficits were observed even after adjusting for height. In this BA population, the magnitude of spine and whole body deficits were significantly greater in older subjects, even when adjusted for confounding variables including gender, pubertal status, race, and height. In other words, bone deficits relative to age in children with BA were due, in part, to factors related to growth and maturation. However, most importantly even taking these factors into account, children with BA have bone deficits of the spine and whole body. 

Our study is one of only a few to investigate bone mass in nonjaundiced subjects with BA. It is unique because of the important use of an appropriate comparison group of healthy subjects and the examination of the effects of body size, race and maturation on bone mineral status, all variables that have previously shown to lead to inaccuracies of DXA interpretations [28]. Despite showing that nonjaundiced participants with BA had depressed bone health, our study has some limitations. Our sample size was small (*n* = 16). Additionally, while our results show a statistically significant decline in BMC with age, our data are cross-sectional and must be confirmed with longitudinal data. The results from this study serve as preliminary data that should be used as the impetus to conduct a larger, longitudinal study of children with BA through age 18 to further investigate the association of BMC with age.

Scant studies have examined rigorously the bone health of nonjaundiced, long-term survivors of BA. Studies that have addressed bone health in this population have often been compromised by extremely small sample populations [29, 30], the use of single beam photon absorptiometry to measure BMD [29, 30], and focus on a younger BA population [30, 31]. In contrast, our sample size was slightly larger than in these previous studies, we used DXA to measure BMC, and our age range allowed for a better indication of the long-term effects of BA on BMC. Further, our study included a more robust control population that permitted adjustment for age, gender, race, maturation, and body size.

It is well known that bone health can be impacted by malabsorption, vitamin K deficiency and vitamin D deficiency [3, 32, 33]. Andrews et al. [32], studied radiographs of a small group of post-Kasai subjects with BA to look for evidence of osteomalacia. They determined that 24% of subjects with “successful” Kasai exhibited radiographic evidence of osteomalacia. However, their results were hindered by the lack of serum vitamin D concentrations and a definition of “successful” Kasai. A more recent study by Bucuvalas et al. [34], examined serum 25–OH vitamin D in nine subjects with cholestatic liver disease. Based on the growing recognition that vitamin D optimization occurs at 25–OH vitamin D concentrations greater than 30 ng/mL [35], 56% of their subjects were suboptimal, similar to the prevalence found in healthy children [36]. Our study found low 25–OH vitamin D levels among subjects with BA with good post-Kasai outcomes; however, data were only available for half of the participants. While these data do not sufficiently support an assertion of vitamin D deficiency, it is possible that vitamin D status may have been one determinant of bone health in our cohort.

Of note is the study performed by Toki et al. [37], which is most closely comparable to ours and substantiates our results. They found two of eight nonjaundiced subjects with BA to have depressed bone health with the presence of normal serum vitamin D, although their definition of normal serum vitamin D status was not given. While they do not specifically address their reference population, we used ours to account for the confounding variables often present in pediatric bone studies [27, 38–41]. The combined results from Toki et al. [37] and our study indicate that compromised bone health may be detected in the absence of severe vitamin D deficiency and therefore warrant future investigations into bone health in nonjaundiced subjects with BA. Another consideration would include exploring any relationships that may exist concerning portal hypertension and bone mineral status.

Optimizing peak bone mass is of utmost importance during childhood and adolescence; it lays the foundation for good bone mineral status through adulthood. Even though past literature has focused on dietary calcium and both dietary and serum vitamin D [10, 34, 40, 42], it is now recognized that inflammation is important and may play a role in bone health [6, 9, 12, 43, 44]. This newest area of research focuses on a group of inflammatory cytokines such as Interleukin-6 [6, 9, 11–13], Interleukin-1 [6, 9, 12, 13], Interleukin-11 [9], and tumor necrosis factor alpha [6, 9, 12, 13], as potential modifiers of bone metabolism that predominantly impair bone formation and promote bone resorption. A recent study by Uchida et al. [22] provided preliminary evidence that various interleukins may affect bone health. However, the study only looked at specific interleukins (Interleukin-6, Interleukin-1 and hepatocyte growth factor). Further studies are needed to evaluate the role of inflammatory factors in bone mineral accretion in a larger sample of children with BA. Most importantly this serves as a bench mark for interventional studies designed to optimize bone health in children.

## 5. Conclusion

Significant bone mineral deficits were identified in a cohort of nonjaundiced BA patients in this study. The focus on a nonjaundiced population, the sample size and the correction for confounding variables makes this a unique study. Pediatric bone health is a priority often overlooked, but is a significant concern because of its long-term implications. It is important to focus on bone health during childhood and adolescence when bone mineral content is increasing rapidly. Achieving optimal peak bone mass is a major determinant of lifelong bone health and prevention of osteoporosis later in life [10]. These data suggest that bone mineral status in children with BA may decline with age during childhood. A larger prospective study is needed to examine bone mineral accretion in this population to identify its determinants, so that effective interventions and strategies can be developed to prevent or treat poor bone health. 

## Figures and Tables

**Table 1:** Subject characteristics between the ages of 3 and 18.

	Biliary atresia		Controls	*P*
*n*	16	11	178	
Age (year) Range	7.3 ± 1.4	8.0 ± 3.6	9.6 ± 3.7	.17
			4.0 to 18.6	
Gender	9 M/7 F	6 M/5 F	76 M/109 F	.38
Race (% African American)	8	10	39	.04
Tanner distribution (**n** at stages 1, 2, 3, 4, 5)	(15, 0, 1, 1, 0)	(9, 0, 1, 1, 0)	(114, 27, 15, 21, 8)	.59
Height **z**-score	−0.31 ± 0.21	−0.11 ± 0.62	0.37 ± 1.10	.14
BMI **z**-score	0.24 ± 0.28	0.18 ± 0.63	0.34 ± 1.10	.64
Weight **z**-score	0.05 ± 0.27	−0.21 ± 0.62
Serum alanine transferase (U/L)^1^	78.4 ± 12.0	56.8 ± 34.6
Serum albumin (g/dL)	3.7 ± 0.2	3.8 ± 0.64
Serum total bilirubin (mg/dL)^2^	2.4 ± 0.9	0.9 ± 0.5
Serum direct bilirubin (mg/dL)^3^	0.24 ± 1.0	0.18 ± 0.13
Serum alkaline phosphatase	267 ± 136	234 ± 108
Serum 25–OH vitamin D (ng/mL)^4^	24.3 ± 5.1	26 ± 5.9
Bone age delay (Years)^5^	0.19 ± 0.38	0.24 ± 1.34

Mean ± SD ^1^Data available for 9 subjects ^2^Data available for 10 subjects ^3^Data available for 10 subjects ^4^Data available for 6 subjects ^5^Data available for 9 subjects

**Table 2:** Multivariable regression models of lumbar spine and whole body BMC in biliary atresia versus control groups adjusting for age, gender, race and height.

		Coef.	Std. Err.	*t*	*P* > |*t*|	*R* ^2^
Lumbar spine BMC						

Model 1	Female gender	0.04	0.03	1.38	0.169	0.82
	Black ethnicity	0.07	0.03	2.16	0.032	
	Age	−1.36	0.39	−3.51	0.001	
	Age^2^	0.56	0.09	6.21	0.000	
	Biliary atresia	−0.14	0.06	−2.21	0.028	
	Intercept	3.38	0.41	8.26	0.000	

Model 2	Female gender	0.04	0.02	1.60	0.112	0.90
	Black ethnicity	0.02	0.02	0.95	0.341	
	Height	2.37	0.23	10.39	0.000	
	Age	−1.58	0.41	−3.84	0.000	
	Age^2^	0.39	0.10	4.08	0.000	
	Tanner stage 2	−0.05	0.04	−1.38	0.169	
	Tanner stage 3	0.05	0.05	0.97	0.333	
	Tanner stage 4	0.14	0.06	2.14	0.034	
	Tanner stage 5	0.25	0.08	2.93	0.004	
	Biliary atresia	−0.12	0.05	−2.58	0.011	
	Intercept	−6.95	0.93	−7.45	0.000	

Model 3	Female gender	0.03	0.02	1.52	0.080	0.90
	Black ethnicity	0.02	0.02	0.94	0.068	
	Height	2.35	0.23	10.39	2.802	
	Age	−1.34	0.43	−3.14	−0.498	
	Age^2^	0.34	0.10	3.43	0.536	
	Tanner stage 2	−0.06	0.04	−1.47	0.019	
	Tanner stage 3	0.06	0.05	1.10	0.166	
	Tanner stage 4	0.15	0.06	2.31	0.272	
	Tanner stage 5	0.26	0.08	3.06	0.420	
	Biliary atresia	0.26	0.22	1.20	0.697	
	Biliary atresia*Age	−0.19	0.11	−1.81	0.017	
	Intercept	−7.14	0.93	−7.66	−5.303	

Whole body BMC						

Model 1	Female gender	0.02	0.03	0.78	0.435	0.85
	Black ethnicity	0.14	0.03	4.38	0.000	
	Age	−0.50	0.40	−1.25	0.214	
	Age^2^	0.39	0.09	4.24	0.000	
	Biliary atresia	−0.12	0.07	−1.90	0.059	
	Intercept	5.93	0.42	14.01	0.000	

Model 2	Female gender	0.02	0.02	0.73	0.467	0.93
	Black ethnicity	0.09	0.02	4.19	0.000	
	Height	2.88	0.21	13.88	0.000	
	Age	−1.65	0.29	−5.67	0.000	
	Age^2^	0.42	0.06	6.59	0.000	
	Biliary atresia	−0.10	0.05	−2.18	0.031	
	Intercept	−5.81	0.90	−6.48	0.000	

Model 3	Female gender	0.01	0.02	0.58	0.563	0.93
	Black ethnicity	0.09	0.02	4.26	0.000	
	Height	2.88	0.21	14.04	0.000	
	Age	−1.44	0.30	−4.71	0.000	
	Age^2^	0.38	0.07	5.64	0.000	
	Biliary atresia	0.35	0.21	1.69	0.094	
	Biliary atresia*Age	−0.22	0.10	−2.21	0.028	
	Intercept	−6.07	0.89	−6.79	0.000	

*All continuous variables (spine and whole body BMC, age, and height) were natural log transformed.

**Table 3:** Spearman correlation of bone mineral content and serologic tests.

	Spine *Z*			Whole body *Z*		
	*Rho*	*n*	*P*	*Rho*	*n*	*P*
Direct bilirubin	−0.64	10	.04	−0.53	10	.11
Total bilirubin	−0.09	10	.81	−0.36	10	.31
ALT	−0.18	9	.65	−0.12	9	.76

## Abbreviations

BA: Biliary atresia
BMC: Bone mineral content
BMD: Bone mineral density
CHOP: Children's Hospital of Philadelphia
DXA: Dual energy x-ray absorptiometry scan
HAZ: Height for age *z*-score
WAZ: Weight for age *z*-score
BMIZ: BMI for age *z*-score
TW2: Tanner-Whitehouse 2
